# Efficacy of radiofrequency ablation for metastatic papillary thyroid cancer with and without initial biochemical complete status

**DOI:** 10.3389/fendo.2022.933931

**Published:** 2022-08-03

**Authors:** Wen-Chieh Chen, Chen-Kai Chou, Yen-Hsiang Chang, Pi-Ling Chiang, Lay-San Lim, Shun-Yu Chi, Sheng-Dean Luo, Wei-Che Lin

**Affiliations:** ^1^ Division of Endocrinology and Metabolism, Department of Internal Medicine, Kaohsiung Chang Gung Memorial Hospital, Kaohsiung, Taiwan; ^2^ Department of Nuclear Medicine, Kaohsiung Chang Gung Memorial Hospital, Kaohsiung, Taiwan; ^3^ Department of Diagnostic Radiology, Kaohsiung Chang Gung Memorial Hospital, Kaohsiung, Taiwan; ^4^ Division of General Surgery and Department of Surgery, Kaohsiung Chang Gung Memorial Hospital, Kaohsiung, Taiwan; ^5^ Department of Otolaryngology, Kaohsiung Chang Gung Memorial Hospital, Kaohsiung, Taiwan

**Keywords:** metastasis thyroid cancer, ultrasound, radiofrequency ablation, tracheal invasion, biochemical incomplete response

## Abstract

**Objective:**

The application of radiofrequency ablation (RFA) for recurrent thyroid cancer has been demonstrated to effectively manage lesions at critical locations, such as abutting the trachea, with limited complications. Comprehensive investigation of both biochemical (B) and structural (S) change after RFA remains limited. We herein present the first single-center experience of RFA for the treatment of locoregional recurrent thyroid cancer in Taiwan.

**Design:**

23 patients were enrolled, and the treatment responses after RFA were divided into four groups (*E*, *S(+)*, *B(+)*, and *SB(+)*), and then compared. The RFA technique, follow-up strategy, changes in pre-and post-operative status, and complications are presented. The volume reduction rate at 1, 3, and 6 months, and the differing responses between lesions abutting/not abutting the trachea are also discussed.

**Results:**

In patients with pre-RFA structural and biochemical incomplete (*SB(+)*) status, presenting with lesion with an initial maximum diameter of >3.2cm, a higher rate of structural incomplete status at the 6-month follow-up was noted in ROC analysis, with a sensitivity of 57% and specificity of 91%. Favorable structural remission after RFA was noted, and 60.9% of patients achieved biochemical complete status. No significant correlation was noted between the trachea-abutted lesion number and complete remission (p= 0.474). No significant difference in RFA efficacy was noted between the lesions abutting/not abutting the trachea.

**Conclusions:**

This retrospective study reveals that RFA can achieve both structural and biochemical improvements for locoregionally recurrent thyroid cancer, with a low complication rate. Nearly half of the patients achieved an excellent response after RFA, while a favorable treatment response can be achieved despite the lesion abutting the trachea, with a mean VRR of 84.74%.

## Introduction

Ultrasound (US)-guided radiofrequency ablation (RFA) is widely applied to thyroid lesions, as an alternative to surgery (1–6). In addition to benign nodules, the application of RFA in differentiated thyroid cancer (DTC) is also being widely investigated (4, 7–9). When metastases are found, a reduction in tumor burden with additional treatments may offer a survival or palliative benefit. The preferred treatment is surgical excision, and multiple factors should be taken into account, including adjacent vital structures, the functional status of the vocal cords, patient comorbidities, and motivation. Localized treatments such as thermal ablation may be beneficial in patients with slowly progressive single or a few metastases (10, 11). The pre-RFA evaluations, techniques, and complication rates differ between RFA for malignant and benign thyroid lesions. As a volume-decreasing technique, previous studies of RFA as applied to metastatic thyroid lesions have primarily focused on structural improvement. We herein present the first report on the efficacy of RFA for locoregionally recurrent DTC in Taiwan. As a single-center study, our investigation began with consideration for both the biochemical (B) and structural (S) status in order to explore the short-term achievements of RFA and changes to pre- and post-RFA status, the associated predicting factors determining complete ablation after RFA, and follow-up strategies. Major complications including hoarseness are discussed, with the aim of providing critical and comprehensive insight into the application of RFA for metastatic DTC.

## Methods

### Patient evaluations

**Figure 1:**
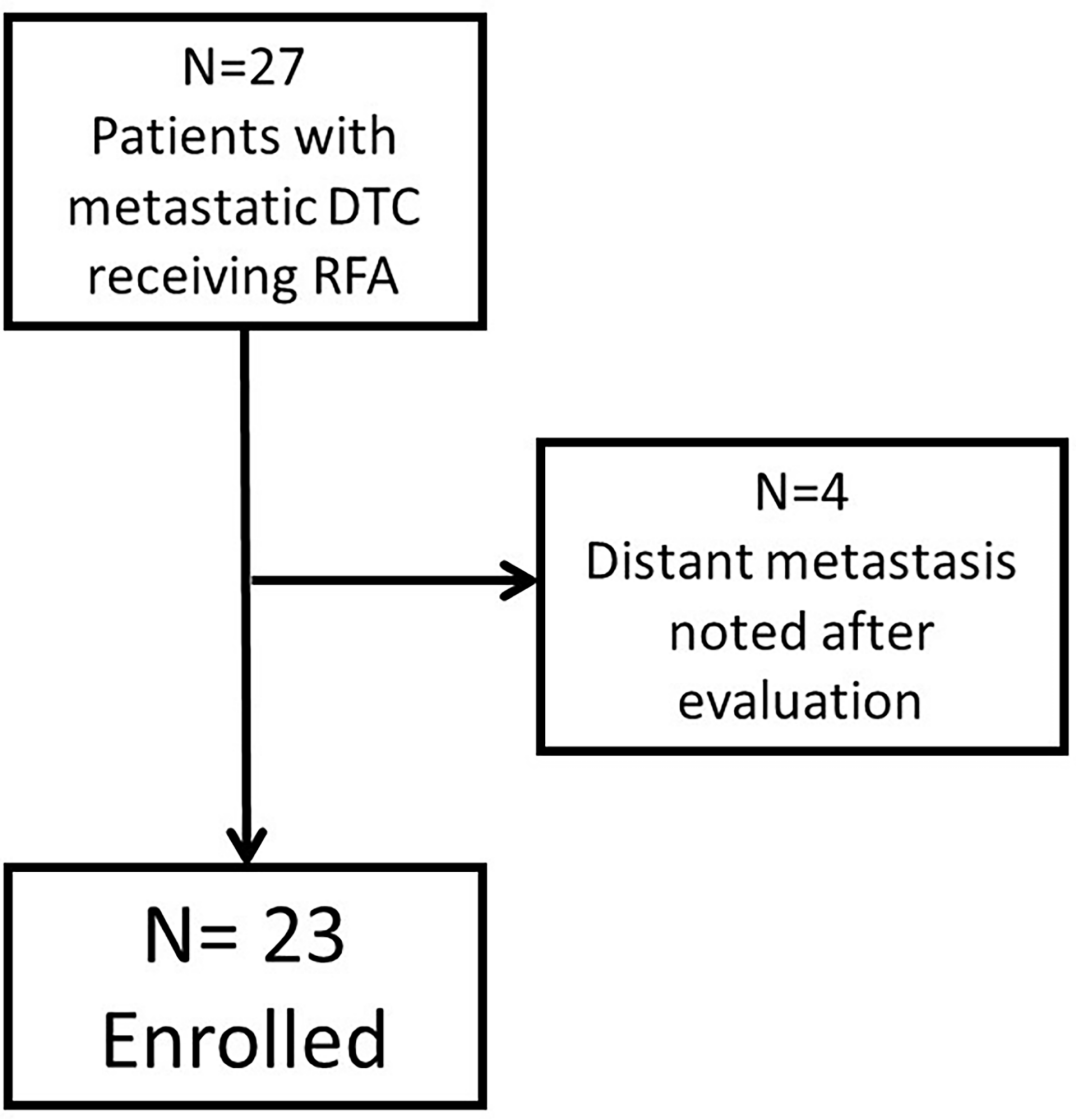
The flowchart of patient enrollment.

A total of 27 patients with recurrent DTC, had received RFA at our institution between Apr. 2017 and Dec. 2020, were evaluated. All patients had previously undergone total thyroidectomy and/or neck lymph node(s) dissection and radioiodine therapy, and were under hormone suppression therapy (thyroxine). After a comprehensive evaluation, 4 patients with distant metastasis (3 with lung and 1 with bone metastasis) were excluded from the study, while 23 patients were enrolled (**Figure 1**). Fine needle aspiration (FNA) cytological findings or thyroglobulin concentration of fine-needle aspirate were used to detect metastases. Recurrent lesions were confirmed by pre-RFA FNA, CNB, or biopsy, and post-ultrasonography (US) and/or computed tomography (CT) evaluations were performed to determine size and location. Data regarding age, sex, previous management, pre-RFA diameter, and volume of the lesions were collected. The lesion volumes were calculated (V = πabc/6; V: volume; a: transverse diameter; b: vertical diameter; c: anteroposterior diameter). Pre-RFA status was also recorded to analyze treatment response. The Tg level was obtained by radioimmunoassay, expressed as nanograms per milliliter. This retrospective study was approved by the Institutional Review Board of our institution.

### RFA procedures

Real-time US-guided RFAs were performed in an outpatient setting. US-guided FNA was performed before RFA to confirm the recurrence status and to guide the RFA procedure. The critical triangle, consisting of the carotid space, trachea, and esophagus was carefully monitored during the procedures. All ablations were performed using an RF generator (Cool-Tip RF system, Covidien; SSP-2000, Taewoong Medical; and M-1004, RF Medical), and an 18-gauge internally cooled electrode (Well-Point RF Electrodes, Taewoong Medical; VIVA, STARmed; Big-Tip, RF Medical; Cool-tip RF system, Radionics, Valleylab) with 5mm or 7mm active tips, depending on the size of tumor and the preference of the radiologist. After para-lesional local anesthesia with 2% lidocaine injected slowly around the tumor, one 18 G/7 cm/5mm or 7 mm RF needle was inserted in the lesion. Ablation was performed using the moving shot technique and completed with a 2-mm (if feasible) safety margin, from the deepest and most remote portion of the lesion, in a unit-by-unit manner (2, 4, 12). For small tumors, the electrode was fixed in the center of the lesion, and not moved during the procedure9. Ablation was initiated with 10–20W of power, and if a transient hyperechoic zone was not formed at the electrode tip within 10 seconds, the power was increased gradually, in 5W to 10W increments, up to 40W. Ablation was terminated when the entire nodule had been changed to transient hyperechoic zones. Hydrodissection was performed to separate the target lesion from the surrounding normal structures, by infusing a 5% dextrose solution into the space between the two sections (13). During the ablation, the patient’s voice and pain were frequently checked. Mild compression at the treatment site was performed for 10-20 minutes, and education regarding self-monitoring for potential complications was given. Follow-ups for US at 1 week, and 1, 3, 6, and 12 months after RFA were scheduled to observe nodule characteristics and volume reduction. CT and/or PET at 6 to 12 months after RFA were also scheduled. Treatment responses and the complications rate were evaluated. All patients were recorded for symptoms of hoarseness and pain before, during and after the RFA procedure.

### RFA response assessment

In accordance with the 2015 ATA guidelines (10), an “indeterminate response” indicates biochemical or structural findings which cannot be classified as either benign or malignant, which is usually an acceptable response. When designing the research method for this study, we referred to the ATA guidelines and chose to use a discriminating definition, not one of “indeterminate response”. The definition of “structural complete” was total remission of the treated lesion(s), with no new lesion found on images. Scar tissue, such as hyper-echoic residual on US or non-enhanced lesion on CT, and reactive lesion of physiological uptake on PET were not included within this range. The definition of “biochemical complete” was basic Tg> 1 ng/ml or stimulated Tg level >10 ng/ml. The status of “structural incomplete” indicated persistent or newly identified locoregional or distant metastases, here abbreviated as *S(+)*. A “biochemical incomplete” response indicated abnormal Tg values in the absence of localizable disease, here abbreviated as *B(+)*. The *SB(+)* abbreviation indicated a patient with both structural and biochemical incomplete status **before** RFA. In summary, all patients in this study were determined as being either pre-RFA *S(+)* or *SB(+)* status.

After RFA, the treatment responses were divided into four groups: excellent (*E*), *S(+)*, *B(+)*, and *SB(+)*. E indicated both biochemical and structural remission, while *SB(+)* indicated both biochemical and structural incomplete responses. *S(+)* indicated existing locoregional remnants, while *B(+)* indicated a biochemical incomplete response, without remnants found.

### Statistical analyses

The demographic data of all subjects is summarized in **Table 1**. To evaluate the effectiveness of the RFA treatment, the following analyses were performed: (1) A patient-based analysis: all patients (n=23), no matter the initial status prior to RFA, were divided into two groups according to the RFA response: structural complete and structural incomplete after the procedure; (2) To further clarify the locoregional effect of RFA, patients with pre-RFA *SB(+)* status (n=18/23) were subdivided into two groups, according to the RFA response: structural complete and structural incomplete after the procedure; (3) Lesion-based analysis: including lesion numbers achieving total remission, the VRR, and a comparison of the VRRs between different post-RFA follow-up periods. The presentation of data included both categorical and continuous variables. Statistical analyses were performed using SPSS Version 23 software (SPSS, Inc.). From the perspective of the patient-based analysis, the correlation between nodule characteristics and treatment response was evaluated. From the perspective of the lesion-based analysis, VRR was analyzed. The characteristics of lesions abutting the trachea were of particular interest and discussion. The chi-square test for evaluation of the categorical variables, and independent-sample t-test for the continuous variables with normal distribution were performed. The Mann-Whitney U test was used for continuous variables with non-normal distribution. P <0.05 was considered significant. The receiver operating characteristic (ROC) curve analysis with Youden’s index was performed to determine the cut-off point of the parameters with regards to post-RFA treatment response.

**Table 1:** Basic characteristics of all patients (n=23).

Pre-RFA status	Total	*S(+)*	*SB(+)*	*P value*
Patient number	23	5(21.7%)	18(78.3%)	–
No. that received RTO	3	0	3	–
No. that received target therapy	1	0	1	–
Sex(male)	6	1	5	–
Age(years old)	53.83 ± 12.92	48.40 ± 13.16	55.33 ± 12.82	0.299
Initial maximum diameter (cm)	1.32 ± 0.79(0.40-3.60)	1.08 ± 0.79(0.7-2.5)	1.39 ± 0.80(0.40-3.60)	0.452
Initial total diameter (cm)	0.95 ± 1.43(0.06-5.77)	0.79 ± 1.41(0.13-3.31)	0.99 ± 1.48(0.06-5.77)	0.788
Initial maximum volume (ml)	1.42 ± 0.75(0.70-3.60)	1.14 ± 0.77(0.7-2.5)	1.49 ± 0.75(0.70-1.60)	0.363
Initial total volume (ml)	1.25 ± 1.62(0.12-5.77)	0.90 ± 1.35(0.23-3.31)	1.35 ± 1.71(1.02-5.77)	0.596
Post RFA change	*→ E*: 10/23(43.5%) *→ S(+)*: 5/23(21.7%) *→ B(+)*: 2/23(8.7%) *→ SB(+)*: 6/23(26.1%)	*→ E*: 2/5(40.0%) *→ S(+)*: 3/5(60.0%)*	*→ E*: 8/18(44.5%) *→ S(+)*: 2/18(11.1%)* *→ SB(+)*: 6/18(33.3%)* *→ B(+)*: 2/18(11.1%)	0.633 0.455 *For those with tumor remained *S(+)*

Treatment response: S(+): structural incomplete, SB(+): structural and biochemical incomplete, B(+): biochemical incomplete, E: excellent

## Results

**Figure 2:**
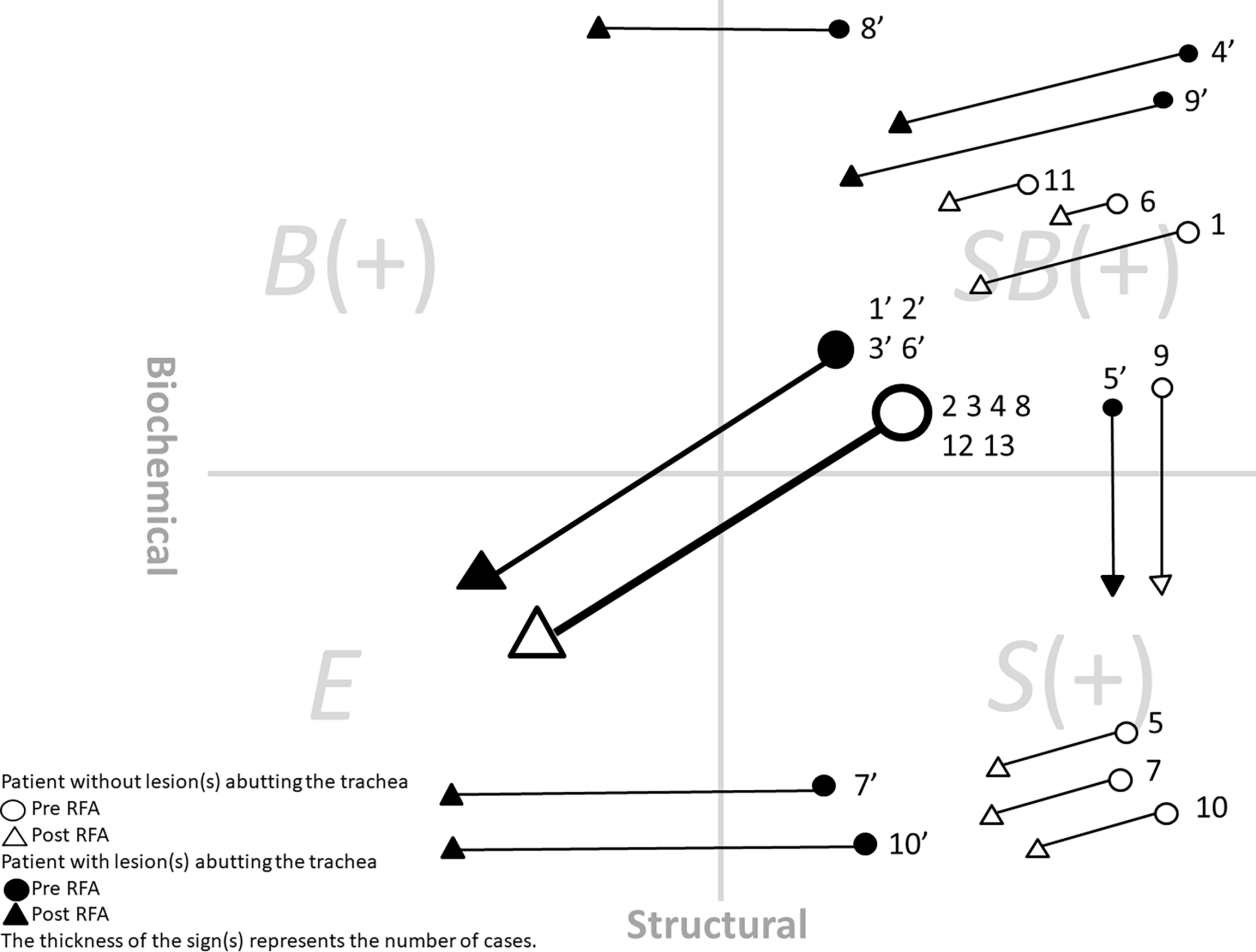
The treatment response and status change after RFA of all 23 patients. White symbols: Patients without lesion abutting the trachea. Black symbols: Patients with lesion abutting the trachea. The four quadrants in the figure indicated SB(+): structural and biochemical incomplete, B(+): biochemical incomplete, E, excellent, and S(+): structural incomplete.

The 23 patients enrolled in the study included 6 males and 17 females, with a mean age of 53.83 ± 12.92 years (range: 31-74) (**Figure 1**). Of these, 22 patients had previously received RAI (95.7%), while 3 received RTO (13.0%, 2 were prior to RFA and 1 was after RFA) (**Table 1**). Initially high post-operative ATA risk stratification (10) with clinical class II-III (14, 15) were noted in all patients. The following results were for a mean time period of 17.70 ± 10.57 months (between 8 and 50 months) after RFA. Of the patients, 43.5% presented an excellent treatment response (E). In the group of patients with pre-RFA *SB(+)* status, 44.4% (8/18) achieved an excellent treatment response (E). To more precisely interpret the individual RFA treatment response, the patients were categorized according to whether or not their lesions had invaded the trachea. Those patients without such lesions were numbered 1 to 13 (indicated by white symbols); while those patients with lesions abutting the trachea were numbered as 1’ to 10’ (indicated by black symbols) (**Figure 2**; **Table 2**). In addition, the characteristics of all patients with structural complete and incomplete responses after RFA were compared (**Table 2**). No significant difference in the number of patients with trachea-invaded lesions nor the lesion number were noted between the two groups. Additionally, no difference in the initial/maximum volume between the two groups was noted.

**Table 2:** Comparison of all patient with structural complete/incomplete response after RFA (n=23).

Treatment response	Structural complete	Structural incomplete	P value
Patient Number	13	10	–
Sex (male)	3(23.08%)	3(30.00%)	0.537
Age (year)	61.0(47-64)	54.0(41.5-62.0)	0.522
Pre-RFA status			
*S(+)*	2(15.4%)	3(30%)	0.400
*SB(+)*	11(84.6%)	7(70%)	–
Patients with Lesions that trachea invasion	7/13 (53.8%)	3/10 (30.0%)	0.237
Total lesions	21	28	–
Lesion no. that invading trachea	9/21 (42.9%)	9/28 (32.1%)	0.318
Initial maximum diameter (cm)	1.44 ± 0.67(0.70-2.80)	1.38 ± 0.88(0.70-3.60)	0.648
Initial total diameter (cm)	2.11 ± 1.41(0.91-6.20)	2.58 ± 0.85(1.20-3.60)	0.077
Initial maximum volume (ml)	1.01 ± 1.16(0.06-3.37)	1.01 ± 1.76(0.13-5.77)	0.605
Initial total volume (ml)	1.23 ± 1.57(0.12-5.52)	1.28 ± 1.77(0.23-5.77)	0.832

With regards to the most complicated patient group, those with pre-RFA *SB(+)* status, we further compared the group that reached structural complete status with the group with structural incomplete status after RFA (**Table 3**). Of the 18 patients with pre-RFA *SB(+)* status, a larger initial total diameter was noted in the group of patients with structural incomplete status after RFA (p= 0.035). Based on the ROC analysis, a metastatic thyroid lesion with an initial maximum diameter of >1.6 cm tended to have a higher rate of structural incomplete outcome. The sensitivity and specificity were 100% and 64%, respectively. When using a diameter of 3.2 cm as the cut-off point, the sensitivity and specificity were 57% and 91%, respectively. The area under curve (AUC 0.805) indicated excellent discrimination. According to the grouping methods applied in **Tables 2** and **Table 3**, the number of trachea-invaded lesions was not significantly correlated to the treatment response, indicating the efficacy of RFA, regardless of whether the lesion had invaded the trachea.

**Table 3:** In patients of pre-RFA *SB(+)* status (n=18), compare the group that reach structural complete and the group remained structural incomplete after RFA.

Treatment response	Structural complete	Structural incomplete	P value
Patient Number	11	7	
Sex (male)	2/11(18.2%)	3/7 (42.9%)	0.272
Age (yr)	61.0(45.0-63.0)	59.0(47.5-63.5)	0.930
Patients with Lesions that trachea invasion	5/11 (45.4%)	3/7 (42.9%)	0.648
Total lesions	19	19	–
Lesion no. that invading trachea	5/19 (26.3%)	5/19 (26.3%)	1.000
Initial maximum diameter (cm)	1.41 ± 0.62(0.70-2.80)	1.63 ± 0.95(0.90-3.60)	0.724
Initial total diameter (cm)	2.14 ± 1.52(0.91-6.20)	2.84 ± 0.73(1.80-3.60)	0.035
Initial maximum volume (ml)	0.87 ± 1.00(0.06-3.37)	1.37 ± 2.03(0.23-5.77)	0.791
Initial total volume (ml)	1.13 ± 1.55(0.12-5.52)	1.69 ± 2.01(0.37-5.77)	0.285

In terms of the biochemical change (non-stimulated Tg), a total of 5 patients remained with a persistently undetectable Tg level after RFA (21.7%). A total 14 patients had a biochemical complete response after RFA (60.9%). For the 18 patients with a pre-RFA biochemical incomplete status, 13 patients had improved Tg levels, while 8 patients had Tg levels which improved to undetectable (44.44%) (**Table 1**). More than half of all patients (13/23, 56.5%) had improved Tg levels after RFA, though with no significant differences in the Tg level change between two follow-up visits (the p values for 1m:3m, 3m:6m, and 1m:6m were 0.060, 0.991 and 0.063, respectively).

Of the 13 patients without lesion(s) abutting the trachea, 3 had pre-RFA hoarseness (3/13, 23.1%), while 1 of those 3 patients presented with a right vocal cord limitation prior to RFA. 3 of the 10 patients with lesion(s) abutting the trachea had pre-RFA hoarseness (3/10, 30.0%). 4 patients experienced new hoarseness during the procedure, 3 of whom recovered within 2-3 months, while 1 remained with persistent hoarseness at the 1-year follow up. No other major complications among the patients were noted.

**Figure 3:**
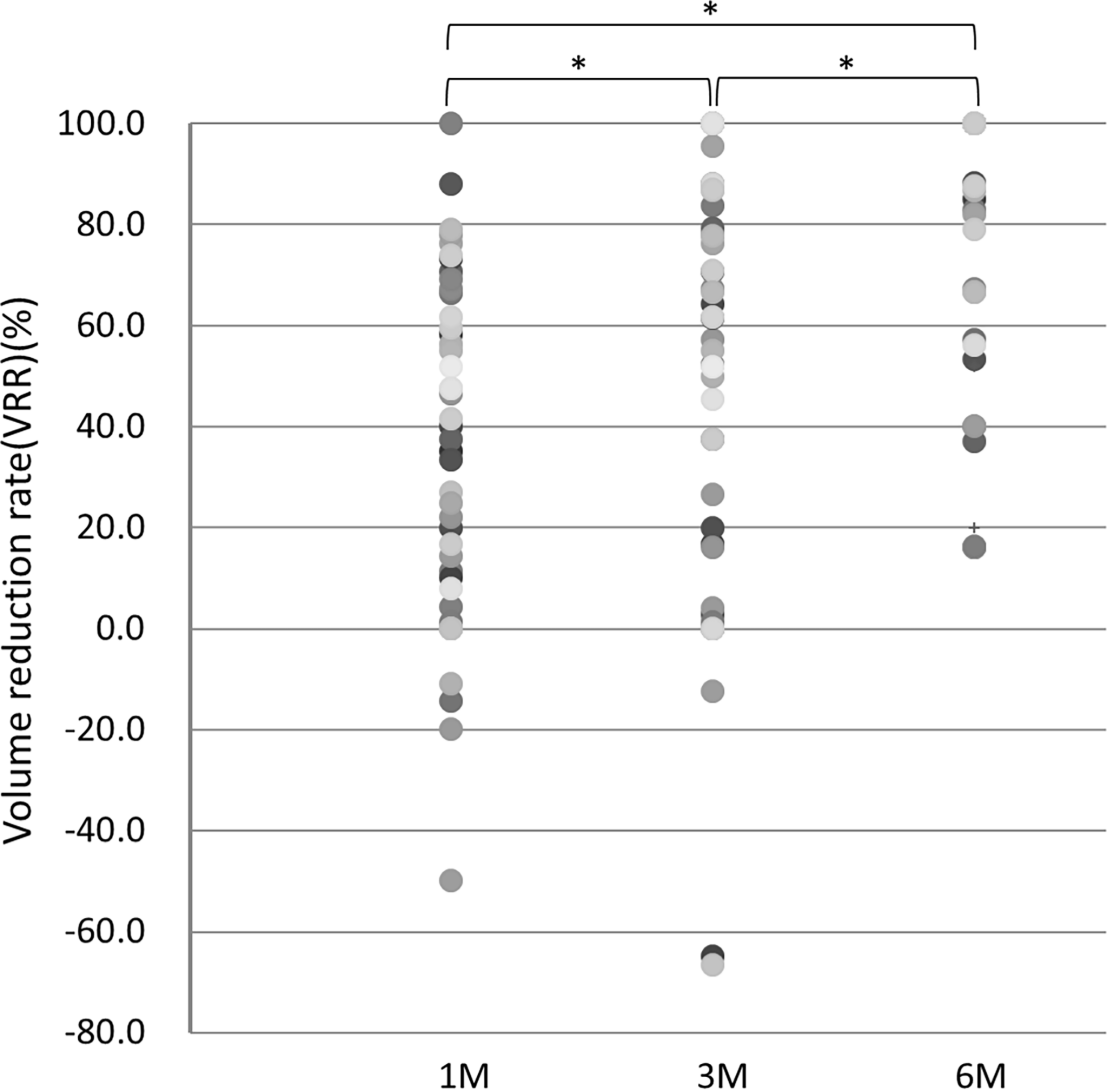
VRR at 1, 3, and 6 months of follow-up. *p value <0.05.

From a lesion-based perspective, significant differences in the VRR were found between each of the two follow-up visits we compared. The p value of VRR change for 1m:3m, 3m:6m, and 1m:6m were 0.031, <0.001, and <0.001, respectively (**Figure 3**). Of the 52 lesions, 29 lesions (55.8%) had completely disappeared at the 6-month follow-up. The mean ( ± SD) VRR at 1, 3, 6 months was 36.56 ± 31.45, 52.97 ± 40.57, and 86.60 ± 22.20%, respectively, indicating ideal structural remission of the metastases within 6 months. In all of the 52 lesions, 29 lesions (55.8%) completely disappeared at follow-up of 6-month period.

Favorable structural remission after RFA was further noted for lesions invading the trachea, with the a mean VRR at 1,3,6, months of 34.29 ± 42.69, 49.31 ± 51.58, and 86.91 ± 19.64%, respectively, while the median VRR of other lesions was 37.24 ± 27.98, 54.30 ± 36.63, and 86.48 ± 23.35%, respectively. A higher VRR of lesions invading the trachea at the 6-month follow-up was noted, though without statistical significance (p=0.955). In summary, 6 of the 29 lesions achieving total structural remission invaded the trachea (20.7%), while 6 of the 23 lesions not achieving total structural remission invaded the trachea (26.1%). No significant correlation was noted between the trachea-invading lesion number and complete remission after RFA (p= 0.474).

## Discussion

### RFA for neck recurrences of thyroid cancer

The first-line management of recurrent thyroid cancer is surgery, while subsequent RAI with thyroid hormone suppression therapy is suggested worldwide (4, 10, 16, 17). However, the risk associated with anesthesia should be considered, while the challenges associated with repeated neck operations increase due to distortion of normal tissue planes, fibrosis, and scar tissue formation. Of note, patients seeking RFA for neck recurrences of thyroid cancer had often previously experienced OP and RAI. Lymphadenectomy results in partial biochemical and structural remission in some of these patients (18), although increased complications as compared to the first operation have been noted (19). It is thus of particular importance to seek appropriate alternatives to an operation, those which may decrease the tumor burden, and achieve both control of locoregional recurrence and a decreased risk of subsequent surgery. The indications of RFA for metastatic DTC primarily include those patients with recurrent thyroid cancer who are at high risk for surgery and those who refuse surgery for various reasons. Minimally invasive treatments (MITs) including US-guided laser, HIFU, and RFA are widely applied to thyroid lesions and should be particularly considered by fragile patients with limited life expectancy, the elderly, those with previous neck dissection or at risk of surgical complications, cases of small size (<20 mm) metastases, and cases with a limited number of lymph node metastases in the latero-cervical compartments (<4 foci) (20–23). Patients with contralateral vocal cord palsy prior to considering management modalities are also candidates for RFA, due to the elevated risk of a compromised airway *via* bilateral vocal cord palsy after repeated surgeries.

In 2001, Dupuy et al. first reported on RFA for recurrent DTC (24): 8 patients with biopsy-proven recurrent DTC underwent RFA (mean size 2.4 cm, range 0.8-4.0 cm); no recurrence at the treatment site was noted at the mean follow-up period of 10.3 months. In the following two decades, several studies regarding the efficacy of RFA for recurrent DTC were subsequently reported (8, 9, 17, 25–28). One retrospective study using a propensity score analysis compared RFA and surgery for the treatment of recurrent thyroid cancer with lesions <2cm. The adjusted 3-year recurrence-free survival rates were comparable for the RFA and reoperation (92.6 vs. 92.2%, P = 0.681) groups, with a similar rate of post-RFA hoarseness. Notably, higher hypocalcemia occurred in the reoperation group (11.6%) but not in the RFA group (0%, P = 0.083) (17). Furthermore, one large retrospective study which included 109 neck lymph node metastases treated with RFA revealed complete responses in 84% at the 38-month follow-up period (29). Meanwhile, a separate retrospective study reported on 42 patients with recurrent DTC who were treated with EA or RFA. While no recurrences in the RFA group after 61 months of follow-up were noted, the study reported that 5 of 21 (23%) metastases had local progression at 4-11 months after EA (25).

Two meta-analyses have further indicated that RFA is an acceptable treatment for locally recurrent thyroid cancer (30, 31). One analysis reported that recurrent tumors completely disappeared in 69% of patients, with a 71.6% serum Tg reduction rate30. Moreover, when it is judged that size reduction may decrease the local tumor burden, relieve compression symptoms, and improve the quality of life for the patient, RFA is indicated even if radiological complete removal is not possible (32). For complete tumor removal, RFA should be applied when image analysis indicates that complete removal by RFA is possible. It is of further importance to note that favorable results have been achieved in recent studies involving RFA undertaken for curative purposes, in which the number of the locally recurrent tumors was less than 3 or 4 per patient and the greatest tumor diameter was smaller than 1.5–2 cm (9, 26, 27).

### Comparing RFA and OP for patients with locally recurrent DTC

Recent studies of RFA treatment for low-risk intrathyroidal PTMCs with long-term follow-up periods (24-60 months) have reported achieving VRRs of 99.8-100% (33, 34). In 2020, Zhang et al. prospectively compared the efficacy of RFA and surgery for patients with isolated intrathyroidal PTMC (35). The surgery group had longer procedure times, longer hospitalizations, and higher treatment costs compared to the RFA group. In addition, 3 patients in the surgery group had complications while none were reported in the RFA group, and all complications occurred in patients with central neck dissection. Such studies indicate that RFA is not inferior to open surgery for carefully-selected PTMC cases in long-term follow-up while reporting relatively higher quality of life and incurring lower costs for the patients (35).

With regards to patients with recurrence, no prospective study has yet directly compared the efficacy of thermal ablation with that of operation. Although, one retrospective study which enrolled 70 patients with locally recurrent thyroid cancer compared those who underwent RFA with those who underwent repeat surgery. Similar recurrence-free survival rates (89.4 vs. 94.5%) were noted in the 6-year follow-up, with no significant differences in the mean post-treatment serum Tg levels. However, hypocalcemia and overall complication rates were significantly higher in the surgery group (7 cases in the RFA vs. 27 cases in the surgery group) (36). Moreover, a study involving RFA of papillary thyroid microcarcinoma found destruction of the tumor tissue, with complete loss of TIFF1 and antimitochondrial antibody expression (37), indicating RFA may be an effective and safe alternative to repeat surgery for recurrent thyroid tumors, with a lower risk of complications.

### Decreased tumor burden

Current thyroid RFA guidelines suggest two treatment strategies for recurrent thyroid cancer, those include curative and palliative treatments. Curative is defined as the treatment of all visible tumors on imaging such as US (4). Whether the lesion is RAI-refractory is not specifically discussed here, since all patients had received RAI prior to RFA. RFA is positioned as the second-line treatment after surgery, while the treatment effectiveness in patients with recurrent thyroid cancer without distant metastasis can be considered close to surgery or RAI. RFA can help these patients effectively reduce tumor volume, even with lesions in a critical location or abutting the trachea. More than half of the lesions (29/52, 55.8%) included in this study had completely disappeared at the 6-month follow-up. In terms of the role lesion size plays in achieving structural complete reduction after RFA, our results indicate that the structural response is related to the initial maximum diameter of the lesion. While in patients with pre-RFA *SB(+)* status and an initial maximum tumor diameter of >3.2 cm, the lesion is less likely to be structural complete after RFA management, with a sensitivity and specificity of 57% and 91%, respectively. With additional future cases and further study, more accurate and insightful results are to be expected.

### Biochemical improvement

Apart from structural changes, the degree of biochemical improvement after RFA also merits attention. In one retrospective study involving longer-term outcomes of RFA for locally recurrent PTC, the mean VRR was 99.5% ± 2.9%, while the mean serum Tg level decreased from 2.55 ± 4.7 to 0.75 ± 1.83 ng/dL (p < 0.001) (38). In addition, a separate meta-analysis found that recurrent tumors completely disappeared in 69% of patients, with a 71.6% serum Tg reduction rate (30). One recent long-term (mean 80 months) follow-up study reported that 91.3% of ablated tumors completely disappeared, with significantly decreased serum Tg levels. Meanwhile, 4 new recurrent tumors and 2 distant metastases were detected during the follow-up period38, indicating that long-term follow-up of Tg levels is necessary after RFA. Additionally, favorable biochemical changes were achieved. In our study graphic presentations was used to intuitively present therapeutic response after RFA (**Figure 2**). 5 patients (21.7%) had persistently undetectable Tg levels after RFA, and 13 (56.5%) had improved Tg levels, further confirming the effectiveness of RFA in reducing the tumor burden.

### Lesions abutting/invading the airway

The initial diameters of lesions abutting the trachea are larger, which may be related to limited treatment having been received before RFA, indicating the importance of early treatment. In 2021, a study by Chung et al. reported on the efficacy of RFA for recurrent DTC, in which all tumors were classified according to their association with the laryngeal structure and trachea. The mean VRR was 81.2% ± 55.7%, with 72.1% of tumors achieving complete remission. The remission rate was the highest in tumors without trachea invasion, followed by tumors forming acute angles, right angles, and those at obtuse angles with the trachea. The poorest remission rate was reported in those tumors with intraluminal tracheal invasion. The overall complication rate was 21.4% (39). A detailed classification of the angle formed by the tumor and trachea was not conducted, which should be the focus of future investigations. In this study, the reason for the higher VRR (though without a significant difference) in the group with the trachea-abutting lesion(s) compared to those without trachea-abutted lesion(s) is unclear. The condition of patients with trachea-abutted lesions was more complicated, thus a combined therapy involving radiotherapy or target therapy may be indicated to achieve a more effective treatment response. Our findings suggest the importance of future investigations involving long-term follow-ups of treatment responses to RFA combined with other management modalities.

### Complications

The low complication rates of thyroid RFA procedures have been confirmed by several guidelines and meta-analyses conducted worldwide (4, 5, 40–42). Major complications associated with RFA include nerve injuries (recurrent laryngeal nerve, cervical sympathetic ganglion, brachial plexus, and spinal accessory nerve), nodule rupture, and permanent hypothyroidism. Minor complications include pain, skin burn, hematoma, and transient thyrotoxicosis or hypothyroidism. Voice change caused by injury to the recurrent laryngeal nerve or vagus nerve is the most common major complication after RFA; the incidence of which has been reported to be 1.45%, with a permanent voice change rate of 0.17% (42, 43). Incidence of voice change is higher for recurrent thyroid cancers (7.95%) than for benign thyroid nodules (0.94%) (44). The variable location of the vagus nerve, especially due to anatomical changes occurring after prior surgery may increase the risk of nerve damage during the RFA procedure (44, 45). To limit the risk of complications, continuous monitoring of the needle tip under US is mandatory during the RFA procedure. Meanwhile, hydrodissection is useful for preventing thermal injury in areas within the vicinity of critical structures.

In our study, 3 of the 10 patients with lesions abutting the trachea had pre-RFA hoarseness (3/10, 30.0%), which is considered to be acceptable. 4 patients experienced new hoarseness during the procedure, which may be related to indirect or direct thermal injury to the nerve, lidocaine infiltration or hematoma on the RLN. Immediate perilesional injection of ice water or intravenous steroids may be helpful in such cases. Meanwhile, 3 of the 4 patients recovered within 3 months after RFA, while 1 remained with persistent hoarseness at the 1-year follow-up. Close cooperation and communication with an otolaryngologist at both pre-and post-RFA status are thus necessary. If unilateral vocal cord palsy is noted prior to the RFA procedure, a re-evaluation of the ablated margin of a peritracheal lesion is crucial. A more conservative treatment plan may be required after a comprehensive discussion with the patient to consider the risk of a compromised airway if bilateral vocal cord palsy occurs. Immediate assessment and intervention are of significant importance to maintaining an acceptable quality of life for patients.

### Limitations and future study

In this study, we focused on the control of locoregional recurrence under the healthcare circumstances at the time, provided with the available data regarding short-term efficacy. In the future, a more systematic study with a longer follow-up period, and the inclusion of other imaging modalities, such as CT, MRI, or PET will be required. In addition, we herein simply noted whether or not the patient experienced pain after RFA; whereas future follow-up studies should include a more detailed quantification of such pain, potentially *via* the visual analog scale (VAS), with data regarding days of pain, management options, and other related factors. The relatively small sample size is also a limitation to the present study. The complex locations of metastatic lesions, such as behind the trachea/bone, laryngeal invasion, the adhesion to critical structures such as the trachea and laryngeal nerve increased the difficulty of accurate post-RFA follow-up. Further imaging modalities, including CT or MRI, may be helpful for soft tissue evaluation (46). Additionally, a longer follow-up with stimulated Tg level in a low-dose whole-body scan would be helpful for an extended evaluation of biochemical change. Furthermore, heterogeneity exists in the current study, without a direct comparison with surgery to more accurately evaluate the relative efficacy of RFA, thus previous retrospective analyses involving such results were discussed (36, 44). An initial static risk assessment after a procedure, followed by continually-modified dynamic risk stratification to re-evaluate the management plan is important (47). The relationship between the degree of initial ATA risk and the outcome of RFA on locoregional recurrence control is currently in development.

## Conclusion

The management of recurrent thyroid cancer is complicated and involves an elevated mortality risk. This study demonstrates that RFA for metastatic thyroid cancer can achieve both structural and biochemical improvements while maintaining a low complication rate. Nearly half of all patients enrolled in our study reached an excellent response (E) status after the RFA procedure; furthermore, favorable treatment response to RFA included those lesions abutting the trachea, with a mean VRR of 84.74 ± 24.44%. In addition, more than half of the patients (56.5%) had improved Tg levels. Among those patients with a pre-RFA structural and biochemical incomplete status, a larger initial total diameter was noted in the group of patients who remained structural incomplete after RFA (p= 0.035). In cases presenting with a metastatic thyroid lesion with an initial maximum diameter of >3.2cm, a higher rate of structural incomplete outcome was noted, with the sensitivity of 57% and specificity of 91%. We hope that the findings provided by this retrospective study of RFA for the treatment of locoregional recurrent thyroid cancer in Taiwan will offer valuable insight into the procedural effectiveness, and assist physicians in more accurately assessing the treatment response to RFA.

## Data availability statement

The raw data supporting the conclusions of this article will be made available by the authors, without undue reservation.

## Ethics statement

The studies involving human participants were reviewed and approved by the Institutional Review Board of Kaohsiung Chang Gung Memorial Hospital. Written informed consent for participation was not required for this study in accordance with the national legislation and the institutional requirements.

## Author contributions

W-CC: collected the data, performed the analysis, wrote the paper. C-KC: contributed analysis tools, performed the analysis. Y-HC: conceived the analysis, contributed data. P-LC: collected the data, contributed data or analysis tools. L-SL: collected the data, wrote the paper. S-YC: contributed data, performed the analysis, S-DL: conceived and designed the analysis, collected the data, performed the analysis. W-CL: designed the analysis, performed the analysis, wrote the paper. All authors contributed to the article and approved the submitted version.

## Conflict of interest

The authors declare that the research was conducted in the absence of any commercial or financial relationships that could be construed as a potential conflict of interest.

## Publisher’s note

All claims expressed in this article are solely those of the authors and do not necessarily represent those of their affiliated organizations, or those of the publisher, the editors and the reviewers. Any product that may be evaluated in this article, or claim that may be made by its manufacturer, is not guaranteed or endorsed by the publisher.
